# Comparison of ^99m^Tc-Labeled Colloid SPECT/CT and Planar Lymphoscintigraphy in Sentinel Lymph Node Detection in Patients with Melanoma: A Meta-Analysis

**DOI:** 10.3390/jcm9061680

**Published:** 2020-06-02

**Authors:** Natale Quartuccio, Ludovico Maria Garau, Annachiara Arnone, Marco Pappalardo, Domenico Rubello, Gaspare Arnone, Gianpiero Manca

**Affiliations:** 1 Nuclear Medicine Unit, A.R.N.A.S. Ospedali Civico, Di Cristina e Benfratelli, 90128 Palermo, Italy; natale.quartuccio84@hotmail.it (N.Q.); annachiara.arnone93@gmail.com (A.A.); gasarno@interfree.it (G.A.); 2 Regional Center of Nuclear Medicine, Hospital University of Pisa, 56126 Pisa, Italy; ludovico.garau@gmail.com (L.M.G.); giamanca@gmail.com (G.M.); 3 Division of Plastic and Reconstructive Surgery, Department of Surgical, Oncological, and Oral Sciences, University of Palermo, 90127 Palermo, Italy; 4 Nuclear Medicine Unit, Santa Maria della Misericordia Hospital, 35100 Rovigo, Italy

**Keywords:** sentinel lymph node, single photon emission/computed tomography, planar lymphoscintigraphy, ^99m^Tc-labeled colloids, melanoma, meta-analysis

## Abstract

We compared the detection rate (DR) for sentinel lymph nodes (SLN_S_), the number of SLNs and the subjects with additional SLNs of single-photon emission computed tomography (SPECT/CT) and planar lymphoscintigraphy (PL) in patients with melanoma. Furthermore, we evaluated the impact of SPECT/CT on surgical plans. Articles containing head-to-head comparisons between SPECT/CT and PL were searched in Pubmed/MEDLINE and Scopus. The literature search was updated until December 31st, 2019. DR was calculated on a per-patient-based analysis; the studies were pooled by their odds ratios (ORs) with a random effects model to assess the significance of difference (*p* < 0.05). The number of additional SLNs (calculated as the relative risk) and pooled proportion of patients with additional SLNs were investigated. The pooled ratio of surgical procedures influenced by the SPECT/CT findings was calculated. Seventeen studies with 1438 patients were eligible for the calculation of DR of SPECT/CT and PL. The average DR was 98.28% (95% confidence interval (95% CI): 97.94–99.19%) for the SPECT/CT and 95.53% (95% CI: 92.55–97.77%) for the PL; OR of 2.31 (95% CI: 1.66–4.18, *p* < 0.001) in favor of the SPECT/CT. There was a relative risk of a higher number of SLNs (1.13) for the SPECT/CT and 17.87% of patients with additional SLNs were detected by SPECT/CT. The average impact of SPECT/CT on surgery resulted in 37.43% of cases. This meta-analysis favored SPECT/CT over PL for the identification of SLNs in patients with melanoma due to a higher DR, reproducibility, number of SLNs depicted, proportion of patients with additional SLNs and the impact on the surgical plan. However, PL remains a good option due to the high values of the DR for SLNs.

## 1. Introduction

The rationale of identifying and removing the sentinel lymph node (SLN), namely the first lymph node in the lymphatic chain draining the primary tumor, relies on the low probability of subsequent metastatic nodes in the case of a lack of cancerous cells in the histological exam performed in the SLN [1,2]. Furthermore, the removal of the SLN avoids the side effects of complete lymph node dissection (CLND) [3,4]. The two main cancers for which SLN biopsy (SLNB) is routinely performed are breast cancer and melanoma [5].

SLNB in patients with melanoma is indicated for T1–T4 stage without clinically evident locoregional or distant metastases [6]. The introduction of SLNB has led to the significant improvement of disease control and better patient outcomes [7]. Reported false negative rates in large cohort trials for patients with melanoma undergoing SLNB range, approximately, from 5% to 20% [8,9,10].

In recent decades, in the efforts of refining the staging, prognosis and treatment of patients with melanoma, liquid biopsy has emerged as an intriguing technique and different methodological approaches have been proposed to detect circulating tumor cells (CTCs), cell-free circulating tumor DNA (ctDNA) or cell-free circulating microRNA (cmiRNA) [11,12,13,14,15]. The detection of CTCs proved to correlate with worse distant metastasis disease-free survival (DFS), reduced recurrence-free survival [14,15,16] and in patients with breast cancer, even to the SLN status [17,18]. Furthermore, a role in the prediction of prognosis and the evaluation of disease progression has been suggested for ctDNA or cmiRNA [13]. These methods may help the detection of tumor dissemination that may bypass the SLNs. Nevertheless, SLNs may also be seen as an “incubator” for subsequent metastases; therefore, the surgical resection of a metastatic SLN would still help halting the disease progression and prevent distant metastases [11].

Whereas liquid biopsy has not been widely translated to the clinical setting, the radionuclide localization of sentinel lymph nodes (SLNs) in patients with melanoma is a well established procedure [19]. Despite the recent introduction in the clinical setting of a new receptor-specific radiotracer (^99m^Tc-tilmanocept) which binds the CD 206 macrophage receptor, ^99m^Tc-labeled colloids are still the most widely used radiotracers for the identification of SLNs [20]. All ^99m^Tc-labeled colloids encompass particles with variable sizes, ranging from 5 to 5000 nm, and share the mechanism of accumulation mediated by the particle size at the level of the SLN, where the particles are phagocytized by the macrophages [21].

Planar lymphoscintigraphy (PL) is currently a routine, simple and reliable procedure, performed in most nuclear medicine departments for the identification of sentinel lymph nodes (SLNs) and lymphatic disorders [22,23]; it consists in an initial dynamic acquisition, followed by early and delayed static acquisitions, completed by an acquisition from the neck to the groin [24].

In recent decades, the use of single-photon emission computed tomography/computed tomography (SPECT/CT) has gained wider diffusion in nuclear medicine departments and increasing evidence has been collected about its superior performance in the detection of SLNs over PL. Whereas a superior overall SLN detection has been reported for the ^99m^Tc-labeled colloid SPECT/CT compared to the PL in cervical cancer [25] in a recent meta-analysis (pooled overall SLN detection odds ratio (OR) of 2.5 (95% CI, 1.2–5.3) in favor of SPECT/CT), such evidence has not been systematically assessed for patients with melanoma. The introduction of hybrid scanners, carrying out scintigraphic and morphological imaging in a one-shot examination, enabled nuclear medicine physicians to provide surgeons with more accurate information regarding SLNs (e.g., location, number and surrounding anatomical structures) compared to the PL. Several individual reports have documented the detections of additional SLNs by the means of SPECT/CT compared to the PL and a meaningful impact of SPECT/CT on surgery.

The aim of this study was to perform a meta-analysis of the head-to-head comparison of the detection rate (DR) of PL and SPECT/CT with the ^99m^Tc-labeled colloids in patients with melanoma. Furthermore, as a secondary aim, we assessed whether the SPECT/CT was able to depict a significantly higher number of SLNs than PL and the proportion of surgical procedures influenced by SPECT/CT findings.

## 2. Materials and Methods

The meta-analysis was conducted in accordance with and in adherence to the PRISMA guidelines (Preferred Reporting Items for Systematic Reviews and Meta-Analyses) [26]. Before starting the literature search, a protocol was developed defining the research question, the search methods, the inclusion criteria, the quality assessment, the data extraction and the statistical analysis.

### 2.1. Literature Search

The PubMed/MEDLINE and Scopus databases were navigated by two researchers to retrieve prospective or retrospective single or multicenter studies, carrying out PL and SPECT/CT in patients with melanoma.

For our primary outcome (detection rate), we selected the articles reporting the DR of PL and SPECT/CT for SLNs (at least 1 lymph node) in patients with melanoma. For our secondary outcomes, we selected articles reporting information on (1) the number of SLNs detected by SPECT/CT and PL, (2) the number of patients with additional SLNs detected by SPECT/CT and/or PL, and (3) the percentage of surgical plans changed on the basis of the SPECT/CT findings. 

The search strings were designed to capture the concepts of melanoma, SLN, SPECT/CT and PL within the title and the abstracts of articles. No date limit or language restriction was applied. The literature search was updated until December 31st, 2019. All the identified references were exported to a reference management software (Endnote v. X7.5, Clarivate Analytics, Philadelphia, PA, United States). 

### 2.2. Study Selection 

An investigator screened the titles and abstracts of the retrieved records. Only original articles were selected. For each outcome of the present meta-analysis, articles from the same authors with a risk of patient overlap were also excluded, selecting only the study with the largest number of patients. Duplicates were identified using Endnote. After excluding duplicates and non-original articles, the full texts of the remaining articles were retrieved to verify the inclusion criteria. 

The full texts were checked to verify the following inclusion criteria: (1) a study cohort or a subset of a minimum of 10 patients with melanoma undergoing both SPECT/CT and PL in the same day for the identification of SLNs; (2) the injection of ^99m^Tc-nanocolloids; and (3) no evidence of other malignancies. Articles in languages other than English were translated by native speakers and included in the meta-analysis. The references of the retrieved articles were also screened for additional studies.

### 2.3. Data Extraction

The data of all included studies in the meta-analysis were independently extracted by two researchers and any disagreement was resolved in a consensus meeting. Bibliographical and technical data extracted from the articles included: the authors, publication year, tracer, tracer activity (expressed in Mega-Becquerel, MBq), number of tracer injections and the approximate timing of SPECT/CT post injection (expressed in minutes). The information regarding melanoma encompassed the anatomical site and the Breslow thickness. 

For the outcome relative to the impact of SPECT/CT on surgical plans, the percentage of surgical approaches influenced by SPECT/CT was based on the surgeon’s judgement retrieved from the articles. The percentage was calculated taking into account the surgical procedures in which the imaging technique determined the following events: (1) a change in location, the size or accuracy of the incision; (2) the localization of an SLN in an accessible anatomical site to the surgery; (3) the SPECT/CT guided the surgeons to SLNs that were undetected on planar images or to SLNs in another basin.

For each article, the following data of the patient sample were retrieved: the number of subjects, sex, age, body max index (BMI), average DR (≥1 SLN) for both the SPECT/CT and PL, along with the absolute number of patients with at least one SLN depicted by SPECT/CT and/or PL, the total number of SLNs detected by SPECT/CT and PL, the number of patients with additional SLNs detected by SPECT/CT or PL and the proportion of patients with changes in surgical management based on the SPECT/CT findings.

### 2.4. Methodological Quality Assessment

The methodological quality of the studies was assessed by an investigator using version 2 of the “Quality Assessment of Diagnostic Accuracy Studies” tool (QUADAS-2) [27], which comprises four domains: patient selection, index test, reference standard, flow and timing. The concerns about the risk of bias or applicability were described as low, high or unclear.

### 2.5. Statistical Analysis

Statistical analysis was carried out using the MedCalc Statistical Software version 19.1.3 (MedCalc Software, Ostend, Belgium; https://www.medcalc.org; 2020). Publication bias was assessed by the visual inspection of the funnel plots. The I^2^ statistic was used to measure the degree of inconsistency across the studies, with I^2^ values of 25%, 50% and 75% representing low, moderate, and high substantial heterogeneity. The interpretation of heterogeneity was carried out at a significance level of *p* = 0.05. The choice of fixed or random effects model to calculate the meta-analytic estimates was made on the basis of the degree of inconsistency, selecting the random effects model in the case of moderate and high substantial heterogeneity. The DR was defined on the basis of the detection of at least one SLN in a single patient. The overall pooled DR was calculated for the SPECT/CT and the PL on a per patient-based analysis and presented using forest plots. In order to assess any statistically significant difference (*p* < 0.05) between the two pooled DRs of the SPECT/CT and PL, the studies were pooled by their odds ratios (ORs) with an inverse variance-weighted random effects model. The number of SLNs detected by SPECT/CT and PL was compared pooling the ORs with an inverse variance-weighted random effects model. The average proportion of patients with additional SLNs detected by each technique compared to the other one and the impact of the SPECT/CT on the surgery were pooled across the studies and presented in the form of percentages in a per patient analysis.

## 3. Results

### 3.1. Literature Search and Eligibility Assessment

**Figure 1:**
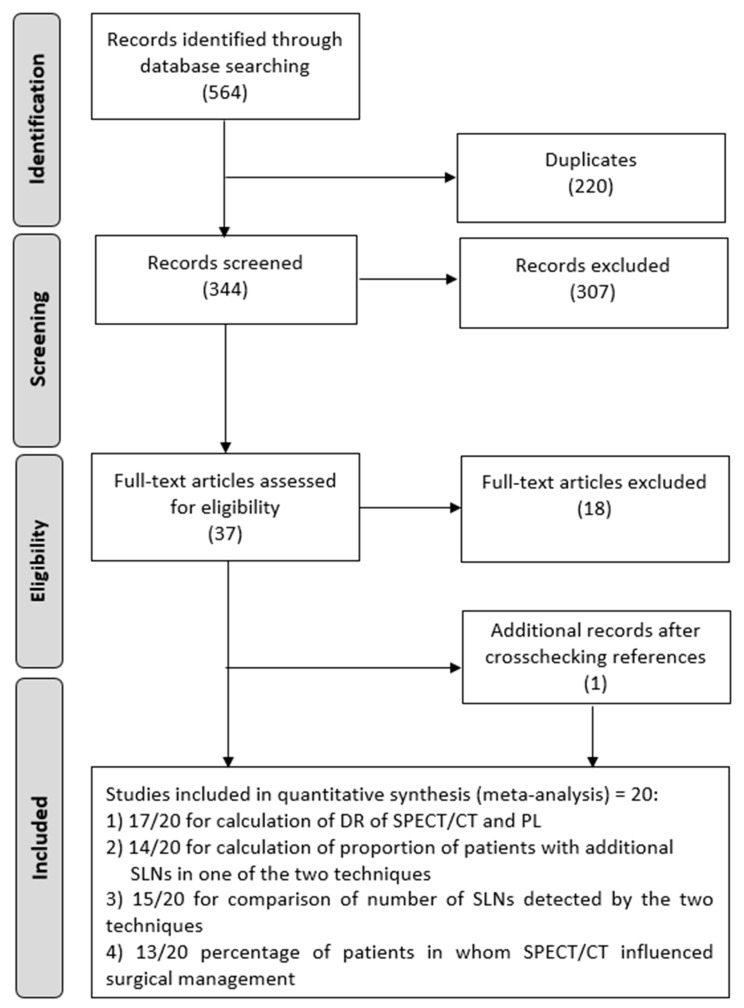
Flow diagram of the literature search. SPECT/CT, single-photon emission computed tomography; PL, planar lymphoscintigraphy; SLNs, sentinel lymph nodes.

The comprehensive computer literature search from the PubMed/MEDLINE and Scopus databases revealed 564 articles (Figure 1). Of these, 220 items were duplicates and excluded. Reviewing titles and abstracts, 307 out of 344 articles were excluded because they were not in the field of interest of this meta-analysis or because they were non-original articles. The full text of the remaining 37 studies was evaluated for the inclusion in the meta-analysis. After checking the full-text, 19 articles were excluded. One additional record was retrieved after cross checking the references. The characteristics of the 20 studies [28,29,30,31,32,33,34,35,36,37,38,39,40,41,42,43,44,45,46,47] selected for the meta-analysis were presented in Table 1. Only 17/19 studies with a total number of 1438 patients were available for the calculation of the pooled DR of the SPECT/CT and PL. Another 14/20 studies were eligible for the calculation of the proportion of patients with additional SLNs in one of the two techniques. Additionally, 15/20 studies were used to compare the number of SLNs detected by the two techniques. Then, 13/20 studies were eligible for the assessment of the average percentage of patients in whom SPECT/CT may have influenced the surgical management. A summary of the data extracted from the studies is available in Table 2. 

**Figure 2:**
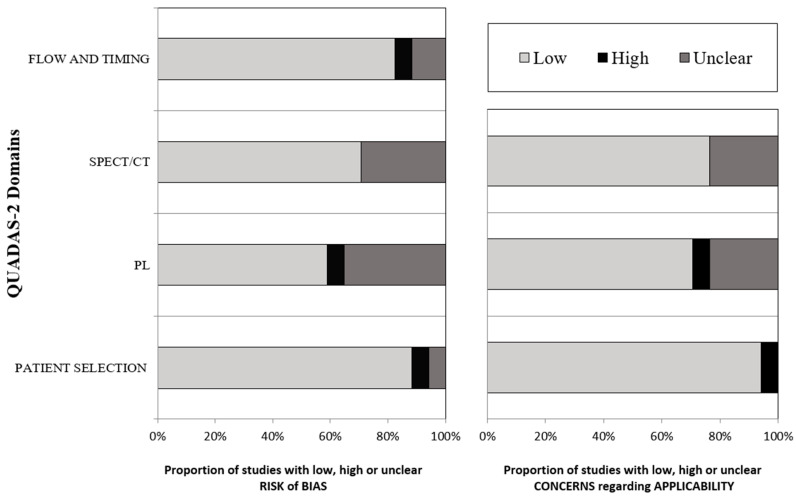
Version 2 of the “Quality Assessment of Diagnostic Accuracy Studies” tool (QUADAS-2) results.

The risk of bias for the 17 studies included in the meta-analysis to calculate the pooled DR (primary outcome) was scored as low by using the QUADAS-2. No publication bias was detected (Figure 2). 

### 3.2. Detection Rate

**Figure 3:**
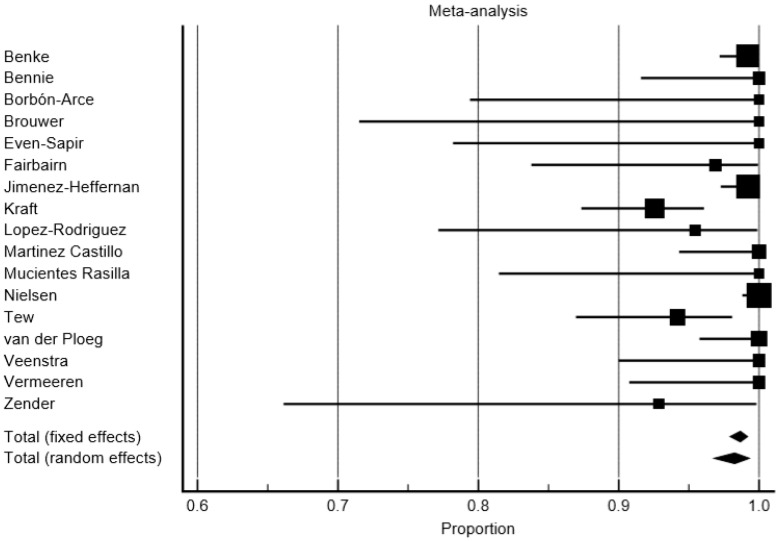
Forest plot of the detection rate (DR) of sentinel lymph nodes (SLNs) on single photon emission computed tomography/computed tomography (SPECT/CT).

**Figure 4:**
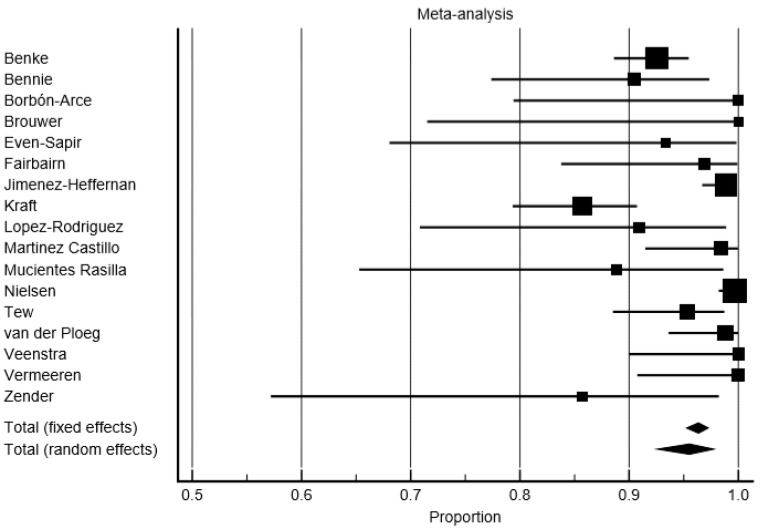
Forest plot of the detection rate (DR) of the sentinel lymph nodes (SLNs) on planar lymphoscintigraphy (PL).

In a per-patient analysis, the overall average DR for the SLNs of SPECT/CT was 98.28% (95% confidence interval (95% CI): 97.94–99.19%), and 95.53% (95% CI: 92.55–97.77%) for PL (Figure 3 and Figure 4). The DR rate of the SPECT/CT for SLNs ranged from 92.5% to 100% across the studies. The DR rate of PL for SLNs ranged from 85.71% to 100%. The consistency of the detection appeared highly heterogeneous for the PL (I^2^ = 78.96%), whereas the heterogeneity was moderate for the SPECT/CT (I^2^ = 62.45%). The significant difference of DR was found: the pooled SLN detection OR of 2.31 (95% CI: 1.66–4.18, *p* < 0.001) in favor of the SPECT/CT. The consistency of a higher DR of SPECT/CT compared to the PL for SLN was found across the study (I^2^ = 0%, CI 95%: 0–39.95%). 

In a per-patient analysis, taking into account only the studies focusing on subjects with a head and neck melanoma (five studies, 101 patients), the pooled DR of the SPECT/CT for SLNs was 97.27% (95% CI: 93.33–99.50%), whereas the DR of the PL was 95.59 (95% CI: 88.58–99.39%). No significant difference of DR was found between the SPECT/CT and PL with an OR of 2.13 in favor of SPECT/CT (95% CI: 0.36–12.50, *p* = 0.4). 

### 3.3. Comparison of Number of SLNs Detected by SPECT/CT and PL

Fifteen articles enrolling 1614 patients reported the number of SLNs detected by SPECT/CT and PL. SPECT/CT depicted a higher number (3587 vs. 3162) of SLNs compared to PL, favoring the use of SPECT/CT with a statistically significant OR of 1.14 (95% CI: 1.06–1.2; *p* < 0.001, random effects model) and a substantial high heterogeneity (I^2^ = 97.17%). In only one article, PL depicted more SLNs than SPECT/CT (144 vs. 143). In the sub-analysis of the studies (n = 5) reporting data on head and neck melanoma (197 patients), there was a statistically significant difference between the number of SLNs detected by the two techniques (427 vs. 487) with an OR of 1.13 in favor of the SPECT/CT (95% CI: 1.06–1.2; *p* < 0.001, random effects model) and moderate heterogeneity (I^2^ = 60.08%).

### 3.4. Average Proportion of Patients with Additional SLNs Detected by SPECT/CT or PL

The assessment of the proportion of patients with additional SLNs depicted by each technique could be extracted from 14 articles. Taking into account a total sample size of 1076, the pooled average proportion of patients in whom SPECT/CT depicted additional SLNs was 17.87% (95% CI: 10.9–26.12%). The inconsistency among articles was high (89.56%); the proportion of patients with additional SLNs depicted by SPECT/CT ranged from 0 (two studies including 11 and 86 patients) to 43.36% (in a study with 113 patients). In one article, PL depicted an additional SLN in one patient that could not be detected by SPECT/CT. In patients with head and neck melanoma (four articles for a total of 85 patients), the pooled percentage of subjects with additional SLNs identified using SPECT/CT was 18.10% (95% CI: 10.9–26.12).

### 3.5. Impact on Surgery of SPECT/CT

The pooled percentage of cases influenced by the use of SPECT/CT (13 studies enrolling 742 patients) was 37.43% (95% CI: 31.95–43.08%). The corresponding percentage of the patients in the studies focusing on head and neck melanoma (four studies including 104 patients) was 49.38% (95% CI: 32.28–66.56%).

## 4. Discussion

In this meta-analysis, we focused on articles comparing SPECT/CT and PL in the same patients rather also including studies with parallel data collection of SPECT/CT and PL in two different patient groups. We believe that this approach allows a more robust investigation of the research question because the patients may serve as their own control.

A very high rate of successful SLNs was detected using PL in institutions with adequate experience [48]. However, the introduction of SPECT/CT in protocols for the identification of SLNs in patients with melanoma has been advocated due to the increasing scientific evidence for a number of its additional advantages over planar imaging [24]. Firstly, SPECT/CT demonstrates a higher DR than PL. In a meta-analysis collecting articles with paired and parallel data in patients with cervical cancer undergoing SPECT/CT (n = 207) and lymphoscintigraphy (n = 208), the pooled median DR was 98.6% for SPECT/CT (range: 92.2–100.0%) and 85.3% for the lymphoscintigraphy (range, 70.0–100.0%) [25]. Whereas, in our meta-analysis, the higher DR of the SPECT/CT compared to the PL was demonstrated in the collection of studies including patients with melanoma located in all regions, the finding was not confirmed when performing a sub-analysis limited to patients with head and neck melanoma (DR: SPECT/CT = 97.27%, PL = 95.59%; OR of 2.13 in favor of SPECT/CT, *p* = 0.4). It is possible to postulate that this discrepancy may depend on the small patient sample derived from the aggregated studies (n = 101 patients, five studies). Despite the marginally larger DR in SPECT/CT, we advocate the use of SPECT/CT in patients with melanoma because a higher DR is correlated to a lower false negative rate and improved DFS. Likewise, the number of detected SLNs per patient has also been demonstrated to be a significant positive prognostic factor for DFS [49].

Further advantages of SPECT/CT over PL include the higher spatial resolution, the precise anatomical localization of the SLN, also defining the relationship to critical anatomical structures [50] and the efficient attenuation correction through exploiting the CT data [25,51].

From our meta-analysis, it can be derived that the results obtained using the SPECT/CT may present higher repeatability than PL, as highlighted by the lower heterogeneity index (I^2^) obtained for the SPECT/CT (I^2^ = 62.45% vs. 78.96%). Another meta-analysis, taking into account patients with papillary thyroid cancer undergoing SLNB, revealed an overall DR of 93% (95% CI: 86–97%) for SPECT and 96% (95% CI: 90–98%) for lymphoscintigraphy; however, while there was a moderate inconsistency for the lymphoscintigraphy data (I^2^ = 68%), there was no heterogeneity for SPECT/CT (I^2^ = 0%) [52].

Our analyses documented a larger number of SLNs detected by SPECT/CT compared to PL (OR: 1.14 in favor). However, these data seem more consistent for patients with head and neck melanoma (I^2^ = 60.08%) across the studies. A higher DR and the larger number of SLNs identified by the SPECT/CT on a head-to-head comparison with PL can theoretically also determine a meaningful impact in surgical decision making. Nevertheless, the preoperative use of SPECT/CT for the identification of SLNs is not only important for the additional number of SLNs but also for the capability of providing anatomical information [21,53]. Furthermore, SPECT/CT may also localize unspecific hot spots that could be mistaken as additional SLNs using only PL [54].

SPECT/CT seems to provide a meaningful impact on surgery, possibly influencing the patient outcome [53]. The large prospective multicenter International Atomic Energy Agency Sentinel Node Trial demonstrated that SPECT/CT had modified the surgical approach in 97 patients with melanoma (37% of the patient population): 41.6%, 39.7%, 33.3% and 30.2% of subjects with head and neck, trunk, lower limb and upper limb lesions, respectively [34]. We found a 37.43% change rate in surgical approaches in patients with melanoma located in all regions and changes in 49.38% of cases for patients with head and neck melanoma. These data, taken together, suggest that factors beyond the additional number of detected SLNs contribute to the change in the surgical plan. A more precise localization of SLNs may lead to a more precise surgical procedure (due to a change in the location, size and accuracy of the incision), facilitating the surgical planning, reducing the morbidity, the duration of surgical operations and the costs [55]. In this regard, Stoffels et al. performed a cost-effectiveness comparative studies between PL alone (254 patients) and PL + SPECT/CT (149 patients) resulting in a mean cost saving when using the second option (€ 710.50), mainly due to a reduction in operative time, shortened hospital stay duration and the more frequent use of local anesthesia [55].

Some limitations should be taken into account in our meta-analysis. The selected studies provided variable sample sizes (ranging from 11 to 307 patients). We cannot be sure whether the DR in studies with a smaller number of patients could be influenced by a lower experience in the identification of SLNs. Another source of bias may derive from the moderate and substantial high heterogeneity we found during the assessment of the studies for the primary and secondary outcomes of the present meta-analysis. Further sources of bias may arise from some methodological differences across the studies including the number of radiotracer injections, total injected activity and the time interval between tracer injection, PL and the execution of SPECT/CT.

## 5. Conclusions

The present meta-analysis provides data that favors the use of SPECT/CT with ^99m^Tc-labeled colloids over PL for the identification of SLNs in patients with melanoma due to the higher DR and reproducibility of the results. However, in institutions where SPECT/CT is not available, PL remains a good option due to its high values of DR for SLNs. SPECT/CT is able to detect additional SLNs compared to PL and may influence surgery in a high percentage of cases. Its impact on surgery may be particularly meaningful in patients with head and neck melanoma.

Although SPECT/CT allows a better surgical planning, there are still cases in which SPECT/CT still does not facilitate the surgical approach and the surgeons do not find an accurate correspondence between the skin marker and the anatomical location of the SLN. In this sense, the widespread of portable gamma-cameras and freehand SPECT may be helpful [56]. Furthermore, future studies with SPECT/CT for the identification of the SLN may focus on the comparison between radiolabeled colloids with fluorescence/hybrid tracers.

## Figures and Tables

**Table 1:** Characteristics of the twenty studies selected for the meta-analysis.

Authors	Year	Country	Tracer	Tracer Activity (MBq)	Tracer Injections	Time Interval (Tracer Injection–SPECT/CT) in min	Anatomical Region	BMI (Mean ± SD)	Breslow Thickness in mm	Number of Patients	M	F	Age (Mean ± SD/Median; Range) in Years
**Benke**	2018	Poland	^99m^Tc-Nanocoll	5–20	2–6	60–180	Trunk	NR	median: 2.0 ± 3.13	255	160	95	median: 61 (17–88)
**Bennie**	2015	South Africa	^99m^Tc-Nanocoll. ^99m^Tc-Sentiscint	NR	4	60	Trunk, upper limb, lower limb	28.5 (n = 23)	NR	42	22	20	mean: M: 50. F:52
**Borbón-Arce**	2014	Spain	^99m^Tc-Nanocoll	Median: 85 (66–158)	3–4	120	Head and neck	NR	mean: 2.7 (1.0–6.0); median: 2.0	16	9	7	mean: 58 (41–77)
**Brouwer**	2012	The Netherlands	ICG–^99m^Tc-nanocolloid	70	4	120	Head and neck	NR	2.7	11	5	6	mean: 54 (32–75)
**Even-Sapir**	2003	Israel	^99m^Tc-rhenium colloid	74	4	NR	Trunk, head and neck, upper limb, lower limb, penis	NR	NR	15 *	*12*	*3*	mean: *57.6 (24–81)*
**Fairbairn**	2013	Scotland	^99m^Tc-Nanocoll	20 or 40	2 or 4	60	Trunk, head and neck, upper limb, lower limb	NR	mean: 2.03 ± 2.26 (0.51–12); median: 1.4	32	12	20	*55* ± 13.66 (17–77)
**Jimenez-Heffernan**	2015	Spain	^99m^Tc-Nanocoll	Mean: 50 ± 27.4	1–6	NR	All regions	NR	0.75–4	262	117	145	53.9 ± 15.2
**Klode #**	2011	Germany	^99m^Tc-Nanocoll	16 or 80	4	120	Head and neck	NR	2.26 (1–7.5); median: 1.7	34	NR	NR	NR
**Kraft #**	2012	Czech Republic	^99m^Tc-Nanocis. ^99m^Tc-Nanocoll, ^99m^Tc-SentiScint, ^99m^Tc-NanoAlbumon	100	4	NR	Trunk, head and neck, upper limb, lower limb	29.4 ± 12.5	NR	113	59	54	mean: 57.6 (11–87)
**Kraft**	2012	Czech Republic	^99m^Tc-Nanocis, ^99m^Tc-Nanocoll, ^99m^Tc-SentiScint, ^99m^Tc-NanoAlbumon.	100	4	NR	All regions	28.4 ± 5.1	NR	161	87	74	57.1 ± 14.8
**Lopez-Rodriguez**	2016	Spain	^99m^Tc-Nanocoll	74	At least 4	NR	Head and neck	NR	mean: 2.96 (1–6)	22	13	9	mean: 55 (24–83)
**Martinez Castillo**	2014	Spain	^99m^Tc-Nanocoll	74	4	NR	Trunk, head and neck, upper limb, lower limb	NR	NR	63	32	31	mean: 55 (25–88)
**Mucientes Rasilla**	2009	Spain	^99m^Tc-nanocolloids (not specificied)	74	4	NR	Trunk, head and neck, upper limb, lower limb	NR	mean: 1.75 ± 1.15 (0.47–4.45)	18	8	10	57.1 ± 20.1 (14–83)
**Nielsen**	2011	Denmark	^99m^Tc-antimony sulphide colloid	40–80	1–2	NR	Trunk, head and neck, upper limb, lower limb	NR	1–4	307	177	130	60 ± 16.9
**Tew**	2017	Australia	^99m^Tc-antimony sulphide colloid	8 or 26 MBq per injection	up to 4	NR	Trunk, head and neck, upper limb, lower limb	NR	NR	86	53	33	mean: 58.8 (22–84)
**Trinh #**	2018	USA	^99m^Tc-filtered sulphur colloid	Mean: 37 ± 10%	NR	NR	Head and neck	NR	NR	118	87	31	58.9 ± 16.7; median: 61 (16–91
**van der Ploeg**	2009	The Netherlands	^99m^Tc-Nanocoll	80	4	120	Trunk, head and neck, upper limb, lower limb	NR	NR	85	NR	NR	mean: 54
**Veenstra**	2012	The Netherlands	^99m^Tc-Nanocoll	69.8 (mean)	NR	120	Trunk, head and neck, upper limb, lower limb	NR	at least 1; or less if Clark level = 4	35	14	21	mean: 60
**Vermeeren**	2011	The Netherlands	^99m^Tc-Nanocoll	71 (mean)	4	120	Head and neck	NR	mean: 2.9 (0.8–7.8); median: 2.2	38	30	8	mean: 53 (24–86)
**Zender**	2014	USA	^99m^Tc-microfiltered sulfur colloid	18–37	NR	NR	Head and neck	NR	mean: 2.68 (1.13–7.0)	14	9	5	mean: 65.43 (31–89)

MBq = Mega-Becquerel; BMI = body mass index; SD = standard deviation; M = male; F = female; NR = not reported; ICG = indocyanine green; # not included in the analysis of primary outcome of the meta-analysis (values of DR not available; DR = detection rate). * extracted from a mixed study population of patients with malignant melanoma and patients with squamous cell carcinoma.

**Table 2:** The data extracted from the twenty studies selected for the meta-analysis.

Authors	Number of Patients	DR of SPECT/CT (%)	DR of PL (%)	Number of SLNs Detected by SPECT/CT	Number of SLNs Detected by PL	% of Patients with Additional SLNs in SPECT/CT	% of Patients with Change in Surgical Plan Based on SPECT/CT Findings
**Benke**	255	99.21	92.54	497	419	15.69	NR
**Bennie**	42	100	90.48	NR	NR	9.52	40.48
**Borbón-Arce**	16	100	100	66	55	NR	NR
**Brouwer**	11	100	100	27	27	0.00	NR
**Even-Sapir**	15 *	100	93.33	NR	NR	NR	35.71
**Fairbairn**	32	96.88	96.88	67	65	18.75	37.50
**Jimenez-Heffernan**	262	99.24	98.85	602	532	20.20	37
**Klode**	34 *	NR	NR	NR	NR	NR	26.47
**Kraft**	113	NA	NA	NA	NA	43.36	NR
**Kraft**	161	92.50	85.70	487	351	NR	NR
**Lopez-Rodriguez**	22	95.45	90.90	NR	NR	27.27	63.63
**Martinez Castillo**	63	100	98.41	266	222	42.86	21.20
**Mucientes Rasilla**	18	100	88.88	31	27	16.67	22.22
**Nielsen**	307	100	99.67	709 **	692 **	NR	NR
**Tew**	86	94.20	95.35	143	144	0.00	39.00
**Trinh**	118	NR	NR	268	234	NR	NR
**van der Ploeg**	85	100	98.82	226	214	8.24	35.29
**Veenstra**	35	100	100	77	69	20.00	31.42
**Vermeeren**	38	100	100	100	94	15.79	55 ***
**Zender**	14	92.86	85.71	21	17	28.57	57.00

DR = detection rate; NA = not applicable; * Extracted as a subset of patients; ** calculated as: mean SLN/patient x number of patients; *** calculated in a subset of 20 patients.
